# What do older patients know about their medication? A cross-sectional, interview-based pilot study

**DOI:** 10.1007/s00228-023-03548-7

**Published:** 2023-08-10

**Authors:** Olaf Krause, Corinna T. Ziemann, Martin Schulze Westhoff, Sebastian Schröder, Benjamin Krichevsky, Stephan Greten, Dirk O. Stichtenoth, Johannes Heck

**Affiliations:** 1 Institute for General Practice and Palliative Care, Hannover Medical School, Hannover, Germany; 2 Center for Geriatric Medicine, DIAKOVERE Henriettenstift, Hannover, Germany; 3 Department of Psychiatry, Social Psychiatry and Psychotherapy, Hannover Medical School, Hannover, Germany; 4 Department of Neurology, Hannover Medical School, Hannover, Germany; 5 Institute for Clinical Pharmacology, Hannover Medical School, Carl-Neuberg-Str. 1, 30625 Hannover, Germany

**Keywords:** Medication knowledge, Older patients, Sick day rules, Medication chart, General practitioner

## Abstract

**Purpose:**

This study sought to analyze the medication knowledge and awareness of medication adjustment options during intercurrent illness (sick day rules) of patients ≥ 70 years treated at a hospital for geriatric medicine in northern Germany.

**Methods:**

The study was designed as a cross-sectional, interview-based pilot study, was approved by the Ethics Committee of Hannover Medical School (No. 10274_BO_K_2022; date of approval: 11 March 2022), and enrolled a convenience sample of 100 patients between May and December 2022.

**Results:**

The median of the average medication knowledge score in the study population (median age 82 years (IQR 75–87); 71% female) was 5 on a scale from 0 to 6 (IQR 3.8–5.6). Women achieved higher average medication knowledge scores than men (median 5.1 (IQR 4–5.6) vs. median 4.3 (IQR 3.6–5.1); *p* = 0.012), and patients < 80 years achieved higher average medication knowledge scores than patients ≥ 80 years (median 5.4 (IQR 4.9–5.7) vs. median 4.3 (IQR 3.2–5.3); *p* < 0.001). Sick day rules were known for only 1.1% of drugs for which sick day rules were applicable. Fifty-two percent of the patients reported that their general practitioner contributed most to their medication knowledge, and 66% considered their daily number of drugs to take adequate.

**Conclusion:**

Our study showed that medication knowledge of older patients was overall satisfying. Awareness of sick day rules, however, was poor. Future studies should evaluate the clinical benefits of sick day rules and ways of better communicating sick day rules to patients. In this regard, general practitioners may play a decisive role.

**Supplementary Information:**

The online version contains supplementary material available at 10.1007/s00228-023-03548-7.

## Introduction

The demographic change in industrialized nations is leading to aging societies and increasing proportions of multimorbid patients who are exposed to polypharmacy, which is commonly defined as the intake of five or more drugs as long-term medication [1]. In Germany, approximately 40% of patients over the age of 65 are exposed to polypharmacy [2].

The ability of patients to understand medication charts and safely and correctly use both prescription medicines and over-the-counter drugs is significantly influenced by their level of health literacy [3]. Advanced age and polypharmacy can lead to deficits in medication knowledge, which may negatively impact on therapy adherence [4] and compromise treatment success [5]. A bicentric study by Freyer and colleagues revealed substantial deficits in medication knowledge among patients of an acute-care hospital and a geriatric rehabilitation clinic [6]. Patients were only able to name approximately half of their discharge medications correctly. Factors influencing knowledge deficits were lack of a medication chart and older age, among others [6]. A study by Krause et al. found that general practitioners (GPs) extensively changed medication regimens of patients discharged from an acute-care geriatric hospital, which may further contribute to deficits in patients’ medication knowledge [7].

Patients who are not familiar with the drugs they are taking, the indications, or options of adjustment during phases of acute illness may not only experience treatment failure—even more serious complications such as adrenal crisis can occur [8]. Insufficient medication knowledge may thus entail an increased use of medical resources [5].

One strategy of dealing with this challenge is communication of so-called sick day rules to patients by healthcare professionals. Sick day rules are instructions to pause or adjust the dosages of certain medications during episodes of acute illness [9–11]. For example, patients with adrenal insufficiency should be instructed to double or triple their daily oral dose of hydrocortisone even during minor illness [12]. Patients taking diuretics may be advised to temporarily discontinue those during phases of diarrhea or vomiting to mitigate the risk of dehydration. For similar reasons, it can be advisable to pause antihypertensive agents such as angiotensin-converting enzyme inhibitors during disease states associated with increased loss of fluid (e.g., febrile infections). If antihypertensive agents were continued without cessation during phases of acute illness, serious complications such as hypotension, dizziness, and falls might result. After a patient’s recovery, these medications can be re-initiated [9].

To date, no generally applicable procedure to inform patients about sick day rules has been established. In Scotland, Medicine Sick Day Rules cards containing information about medication adjustments during episodes of acute illness were introduced by the National Health Service (NHS) Highland in 2013 [13]. According to NHS Highland, medicines that should be paused during phases of intercurrent illness are angiotensin-converting enzyme inhibitors, angiotensin-receptor blockers, non-steroidal anti-inflammatory drugs, diuretics, and metformin [13]. Although this initiative by NHS Highland temporally correlated with a decrease in hospital admissions [14, 15], suggesting potential therapeutic benefits, sick day rules cards have neither been standardized in the United Kingdom nor internationally. Besides the medications proposed by NHS Highland, Diabetes Canada added direct renin inhibitors (e.g., aliskiren), sulfonylureas (e.g., gliclazide, glimepiride, glyburide), and sodium–glucose cotransporter 2 inhibitors to their Sick-Day Medication List of drugs to withhold during acute illness and/or states of dehydration [16]. Sodium–glucose cotransporter 2 inhibitors should be paused during intercurrent illness to reduce the risk of potentially life-threatening diabetic ketoacidosis [17]. A review article by Keller also mentioned insulins (e.g., insulin aspart, insulin lispro, insulin degludec, and insulin glargine) as examples of drugs for which sick day rules may be communicated to patients [9].

Morris and colleagues reported that GPs and pharmacists considered sick day rules useful; however, GPs and pharmacists disagreed about who should ultimately be responsible to inform patients about sick day rules [10].

In the present study, we analyzed the medication knowledge and awareness of adjustment options (i.e., sick day rules) of patients aged ≥ 70 years who were treated at a hospital for geriatric medicine in northern Germany. We investigated potential influences of age or sex on medication knowledge and whether patients’ medication knowledge differed between drug groups.

## Methods

### Study design

The study was designed as a cross-sectional, interview-based pilot study. It was planned to enroll a convenience sample of 100 patients. To this end, patients treated at the Center for Geriatric Medicine (Zentrum für Medizin im Alter), DIAKOVERE Henriettenstift, Hannover, Germany between May and December 2022 were screened for eligibility (see section “Eligibility criteria”) and asked by one of the joint first authors (OK or CTZ) to participate in the study. Patients willing to participate in the study were informed about the aims of the study, and written informed consent was obtained. Patients who met all eligibility criteria and who had provided written informed consent were interviewed by CTZ using a questionnaire specifically designed for this study (see section “Study questionnaire”). Patients were allowed to use their medication chart during the interview. Patients’ relatives—if present at the time of the interview—were allowed to stay and support the study participants during the interview, as this situation better reflected real-world conditions.

### Study questionnaire

To address the research questions of this study, a questionnaire was devised by the study authors (Supplementary Material 1 (English version) and Supplementary Material 2 (German version)). The questionnaire was used by CTZ during the interviews and addressed the following medication-related topics: drug name; indication; dosage; and frequency of application.

These medication-related topics were investigated for each drug in the medication charts of the study participants. One point was achievable for the categories drug name and frequency of application, while up to two points were achievable for the categories indication and dosage. The reason for the different maximum scores in the categories drug name and frequency of application (one point) as opposed to the categories indication and dosage (two points) was that more nuanced answers were expected for the latter two categories, requiring a more differentiated scoring system. For each drug, the achieved points were summed up to yield the medication knowledge score (range 0–6, with higher scores indicating better medication knowledge). To allow for meaningful comparisons between patients taking different numbers of drugs, the medication knowledge scores of all drugs of a patient were summed up and subsequently divided by the number of drugs the patient was taking, yielding the average medication knowledge score (range 0–6, with higher scores indicating better medication knowledge). Examples of patient answers and corresponding ratings are provided in Table 1. For instance, a patient who answered that she took “Xarelto, for blood thinning, one tablet in the morning” would achieve 4 out of 6 points for this drug (a perfect, 6-point answer in this example would have been: “Xarelto, for blood thinning to prevent a stroke, one 20-mg tablet in the morning”). Correctness of answers was evaluated based on the medication chart provided to patients by their GP. If patients did not have a GP, the medication chart from the Center for Geriatric Medicine was used to assess answer correctness.

**Table 1:** Examples of patient answers and corresponding ratings according to the study questionnaire (Supplementary Material 1 and Supplementary Material 2) for six frequently used medication classes

**Medication-related topic**	**Description of answer qualities for 0, 1, or 2 points, and examples of patient answers for the respective categories**		
	**0 points**	**1 point**	**2 points**
**Drug name**^**a**^	The name of the drug is not known by the patient	The drug can be named correctly by the patient (both generic names and brand names are accepted). Minor errors (e.g., wrong pronunciation, omitted or transposed syllables) are tolerated	n.a.
Analgesics	Drug name not known	Metamizole; Novalgin^®^	n.a.
Antihypertensives		Ramipril; Delix^®^	
Diuretics		Furosemide; Lasix^®^	
Oral anticoagulants		Rivaroxaban; Xarelto^®^; phenprocoumon; Marcumar^®^	
Oral antidiabetics		Metformin; metorfmin [sic]	
Proton pump inhibitors		Pantoprazole; prantopazole [sic]; Pantozol^®^	
**Indication**^**a**^	The indication for the drug is not known by the patient	The indication for the drug can be attributed to an organ (system) by the patient	The indication for the drug can be stated precisely by the patient
Analgesics	Indication not known	“Against pain”	“Pain after surgery”, “pain after a fall”
Antihypertensives		“For my heart”; “for the circulation”	“Against high blood pressure”
Diuretics		“Water pill”; “to urinate”; “to remove water from my body”	Edema; heart failure
Oral anticoagulants		“For blood thinning”	“For blood thinning to prevent a stroke”; “thrombosis”; “atrial fibrillation”
Oral antidiabetics		“For my blood sugar”; “sugar pill”	Diabetes (mellitus)
Proton pump inhibitors		“For my stomach”	“For stomach protection”; “heartburn”; “against gastric acid”
**Dosage**^**a**^	The dosage of the drug is not known by the patient	The dosage is known semiquantitatively by the patient, that is, in shares of one tablet	The dosage is known quantitatively by the patient. Answers without precise units of measurement (e.g., gram, milligram, etc.) are tolerated
Analgesics	Dosage not known	½ tablet; ¼ tablet	Metamizole 500 mg; Novalgin^®^ 500
Antihypertensives			Ramipril 10; Delix^®^ 10 mg
Diuretics			Furosemide 20 mg; Lasix^®^ 20
Oral anticoagulants			Rivaroxaban 20; Xarelto^®^ 20 mg
Oral antidiabetics			Metformin 1 g; metformin 500
Proton pump inhibitors			Pantozol^®^ 20, Pantoprazol 20 mg
**Frequency of application**^**a**^	The frequency of application of the drug is not known by the patient	The frequency of application is known by the patient	n.a.
Analgesics	Frequency of application not known	Once daily; twice daily; “1–0–1”; “in the morning and evening”	n.a.
Antihypertensives			
Diuretics			
Oral anticoagulants			
Oral antidiabetics			
Proton pump inhibitors			
**Sick day rule**^**b**^	The sick day rule is not known by the patient	The patient is familiar with the sick day rule	n.a.
Analgesics	Sick day rule not known	Stop taking diclofenac during acute gastroenteritis	n.a.
Antihypertensives		Pause ramipril while suffering from diarrhea	
Diuretics		Discontinue torasemide while vomiting	
Oral anticoagulants	n.a.		
Oral antidiabetics	Sick day rule not known	Pause metformin during acute illness	n.a.
Proton pump inhibitors	n.a.		

Abbreviations: *n.a.* not applicable

^a^The achieved points for these medication-related topics were summed up for each drug to yield the medication knowledge score (range 0–6, with higher scores indicating better medication knowledge)

^b^Knowledge of sick day rules was analyzed separately, that is, the achieved points were not integrated into the medication knowledge score

Knowledge of sick day rules was investigated separately by asking patients for each drug of their medication chart to which a sick day rule was applicable (e.g., ACE inhibitors, NSAIDs, diuretics, etc.): “Is there anything to observe for this drug in case of acute illness?”. Examples of patient answers on sick day rules and corresponding ratings are also showcased in Table 1. Of note, over-the-counter (OTC) preparations were excluded from analysis due to the absence of a prescribing physician who could have possibly communicated sick day rules to the patient.

In addition, patients’ opinion about their daily number of drugs was evaluated on a 5-point Likert scale: 1 = “too few”; 2 = “rather too few”; 3 = “adequate number”; 4 = “rather too many”; 5 = “too many”.

Finally, patients were asked who or what contributed most to their medication knowledge, using a predefined 8-item list of answer options (single choice): pharmacy; general practitioner; medical specialist; partner/spouse, relatives, friends; television; the press, magazines; the Internet; or other.

### Drugs and drug classes for which sick days rules were considered applicable

Based on our review of the literature [9, 12, 13, 16, 17] and pathophysiological considerations, we decided that, for the purpose of this study, sick day rules were applicable to antihypertensive agents (e.g., angiotensin-converting enzyme inhibitors, dihydropyridine calcium channel blockers, diuretics, etc.), laxatives, insulins, oral antidiabetic agents (e.g., metformin, sulfonylureas, sodium–glucose cotransporter 2 inhibitors, etc.), methotrexate, glucocorticosteroids, and non-steroidal anti-inflammatory drugs.

### Pretest

Prior to the start of the study, a pretest with three randomly selected patients was conducted, which confirmed the applicability of the study questionnaire under real-world conditions and the feasibility of the study as a whole. No modifications of the questionnaire were necessary after the pretest.

### Eligibility criteria

Patients were eligible for enrollment in the study (i) if they were treated at the Center for Geriatric Medicine, DIAKOVERE Henriettenstift, Hannover, Germany, between May and December 2022 as inpatients or outpatients (in the hospital’s day clinic); (ii) if they were aged ≥ 70 years; (iii) if they had been regularly taking at least five different drugs for at least the past three months; and (iv) if they were able to provide written informed consent.

### Drug classification

Drugs were classified according to the Anatomical Therapeutic Chemical (ATC) Classification for Germany, version 2022 [18]. For statistical analyses, first-level ATC codes were used.

### Statistical analyses

All statistical analyses were performed with IBM SPSS Statistics for Windows, version 28 (Armonk, New York, USA). Quantitative variables were tested for normal distribution with the Shapiro–Wilk test. Due to skewed distribution, quantitative variables are depicted as medians with interquartile ranges (IQRs). For quantitative variables, the Mann–Whitney *U* test or Kruskal–Wallis test was used to investigate potential differences between two groups or ≥ three groups, respectively. Categorical variables are reported as absolute and relative frequencies. *P *values (two-sided) < 0.05 were considered statistically significant. Due to the exploratory character of our study, no adjustments for multiple testing were conducted.

## Results

### Study population and interview characteristics

**Fig. 1:**
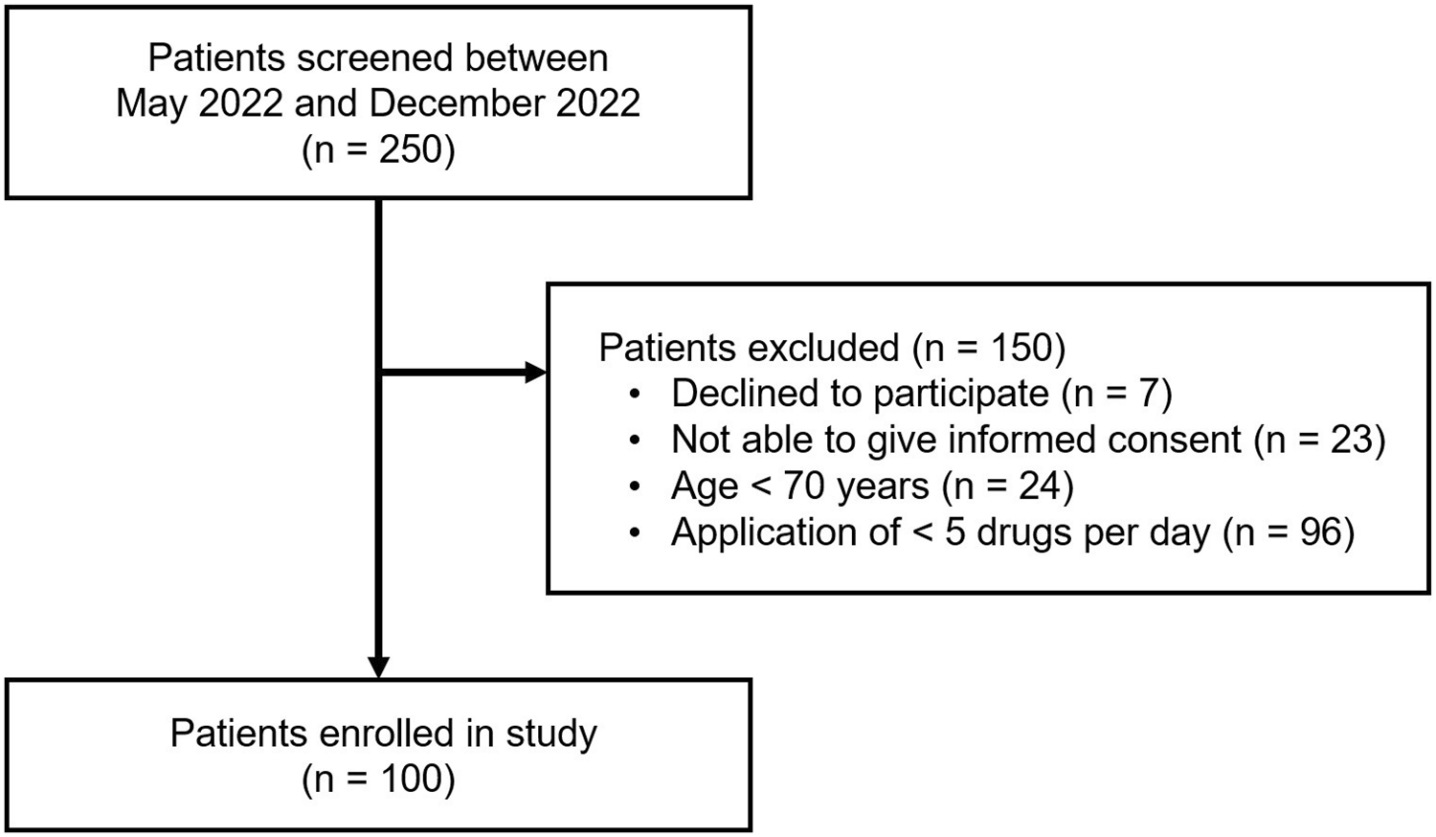
Flow of participants

250 patients were screened between May and December 2022 to enroll the pre-planned convenience sample of 100 participants (Fig. 1). The most frequent reason for exclusion was use of less than five drugs per day. The median age in the study cohort (n = 100) was 82 years (IQR 75–87 years; range 70–96 years) and 71% of the patients were female. Forty-one percent of the patients were < 80 years, while 59% were ≥ 80 years.

Patients took a median of 8 drugs per day (IQR 6–10 drugs; range 5–17 drugs). The number of drugs per day did not differ statistically significantly between men and women (median 8 drugs (IQR 7–9.5 drugs) vs. median 8 drugs (IQR 6–10 drugs); *p* = 0.863), nor between younger (i.e., < 80 years) and older (i.e., ≥ 80 years) patients (median 7 drugs (IQR 6–10 drugs) vs. median 8 drugs (IQR 7–10 drugs); *p* = 0.131).

The median duration of the interviews was 24.5 min (IQR 20–30 min; range 15–60 min), and 95% of the patients used their medication chart as support. Patients’ relatives were present in 8% of the interviews.

### Average medication knowledge score

The median of the average medication knowledge score in the total study population was 5 (IQR 3.8–5.6; range 0.9–6). Women achieved higher average medication knowledge scores than men (median 5.1 (IQR 4–5.6) vs. median 4.3 (IQR 3.6–5.1); *p* = 0.012), and patients < 80 years achieved higher average medication knowledge scores than patients ≥ 80 years (median 5.4 (IQR 4.9–5.7) vs. median 4.3 (IQR 3.2–5.3); *p* < 0.001).

### Comparison of drug groups

To investigate potential differences in patients’ medication knowledge about different drug groups, the four most frequently used drug groups in the study population (i.e., ATC groups A (alimentary tract and metabolism), B (blood and blood forming organs), C (cardiovascular system), and N (nervous system); Table 2) and the remaining drugs (summarized as “other”) were compared. The latter group comprised ATC groups D (dermatologicals), G (genitourinary system and sex hormones), H (systemic hormonal preparations, excluding sex hormones and insulins), J (antiinfectives for systemic use), L (antineoplastic and immunomodulating agents), M (musculoskeletal system), P (antiparasitic products, insecticides, and repellents), R (respiratory system), S (sensory organs), and V (various). Patients’ medication knowledge scores did not differ between ATC groups A (median 5 (IQR 4–6)), B (median 5 (IQR 4–6)), C (median 5 (IQR 4–6)), N (median 6 (IQR 4–6)), and the group of “other” drugs (median 5 (IQR 4–6)) (*p* = 0.688).

**Table 2:** Categorization of drugs (n = 819) taken by the study population according to the World Health Organization’s Anatomical Therapeutic Chemical (ATC) classification system

**Anatomical Therapeutic Chemical (ATC) group (1**^**st**^** level)**	**n**	**%**
A (Alimentary tract and metabolism)	177	21.6
B (Blood and blood forming organs)	96	11.7
C (Cardiovascular system)	331	40.4
D (Dermatologicals)	1	0.1
G (Genitourinary system and sex hormones)	8	1.0
H (Systemic hormonal preparations, excluding sex hormones and insulins)	43	5.3
J (Antiinfectives for systemic use)	1	0.1
L (Antineoplastic and immunomodulating agents)	3	0.4
M (Musculoskeletal system)	27	3.3
N (Nervous system)	107	13.1
P (Antiparasitic products, insecticides, and repellents)	1	0.1
R (Respiratory system)	13	1.6
S (Sensory organs)	6	0.7
V (Various)	5	0.6

### Knowledge of sick day rules

Sick day rules were applicable to approximately one-third of drugs used by the study population (33.1%; 271/819). Of note, sick day rules were known for only 3 of these 271 drugs (1.1%). The 3 drugs for which sick day rules were known (candesartan, furosemide, methotrexate) were taken by 3 different patients. For the remaining 268 drugs (98.9%), patients were not aware that sick day rules existed.

### Patients’ opinion about their daily number of drugs to take

**Fig. 2:**
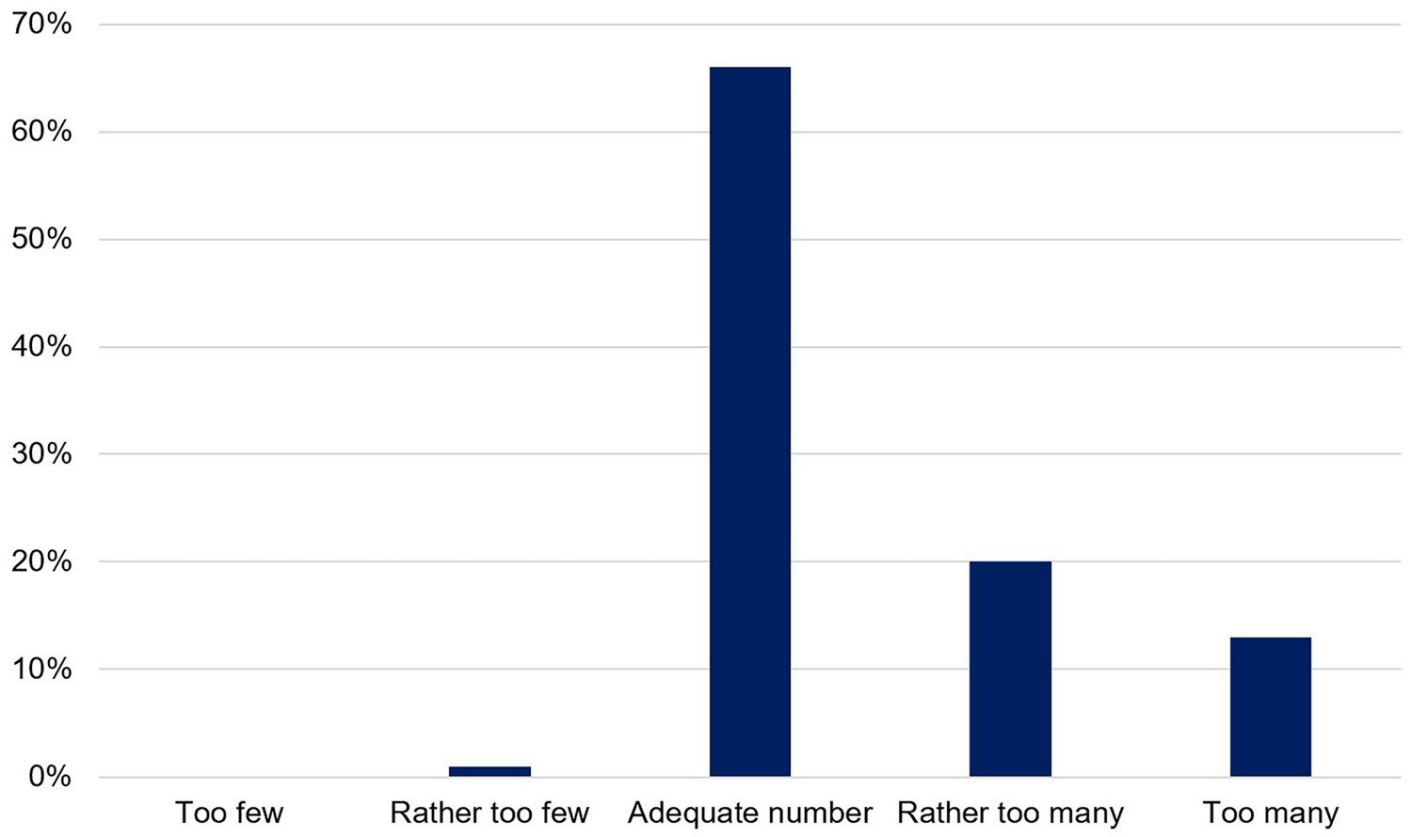
Patients’ opinion about their daily number of drugs to take

Two-thirds (66%) of the patients considered their daily number of drugs to take “adequate” (Fig. 2). Every fifth patient (20%) and 13% of the patients found that they had to take “rather too many” or “too many” drugs per day, respectively.

### Contribution to medication knowledge

**Fig. 3:**
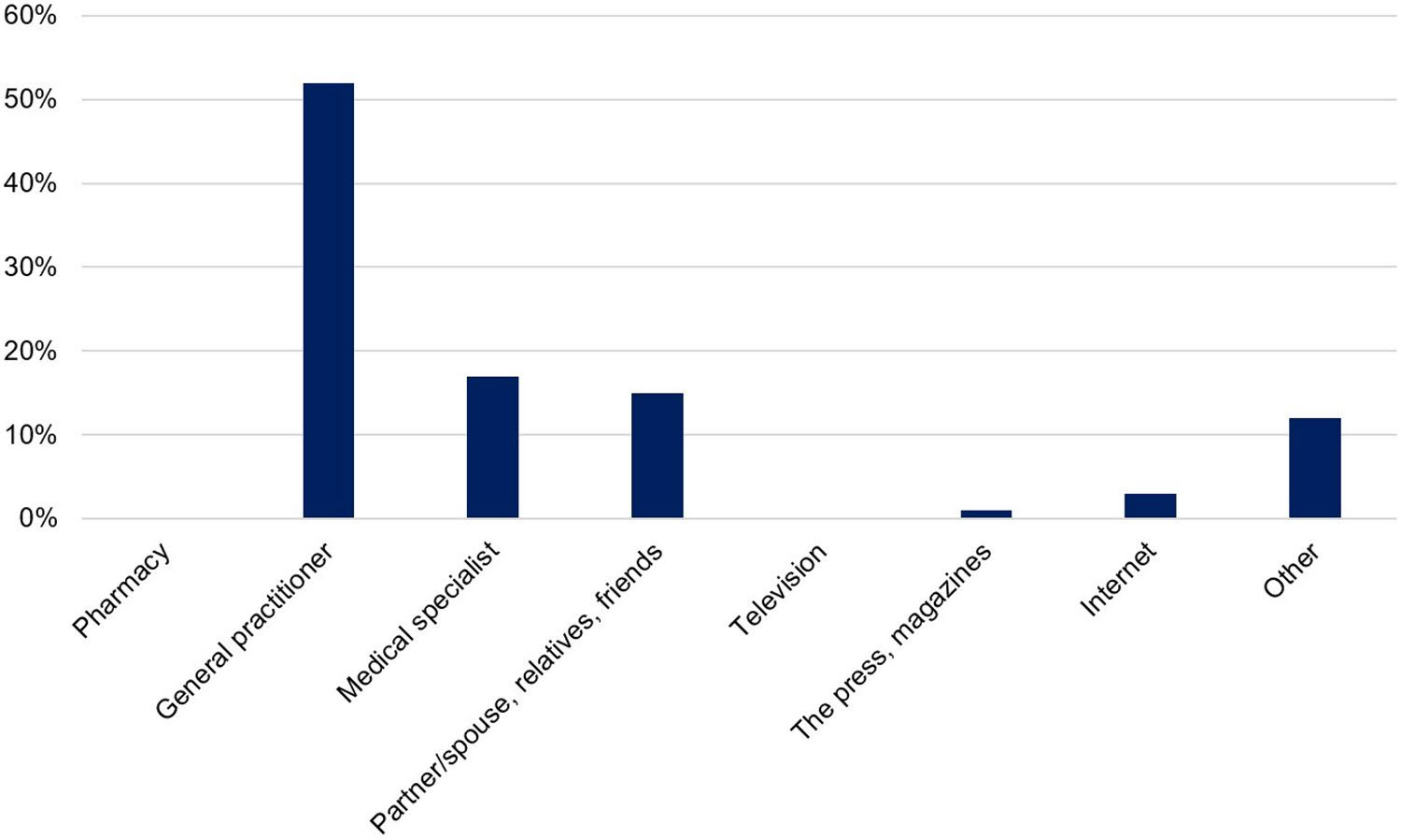
Contributors to patients’ medication knowledge. Based on a predefined 8-item list, patients were asked who or what contributed most to their medication knowledge

More than half of the patients (52%) reported that their general practitioner contributed most to their medication knowledge, with medical specialists (e.g., cardiologists, neurologists, etc.) occupying the second rank (17%) (Fig. 3). Astonishingly, the media only played a minor role in contributing to patients’ medication knowledge (television: 0%; the press, magazines: 1%; the Internet: 3%). Twelve percent of the patients used “other” information sources. When specifically asked about those “other” information sources, all twelve patients named the package insert.

## Discussion

Our study provided three main results. First, female sex and younger age (< 80 years) were associated with higher medication knowledge. Second, two-thirds of the patients considered their number of drugs to take per day appropriate, suggesting a high level of trust in prescribers, especially general practitioners (GPs), who also contributed most to patients’ medication knowledge in our study. Third, sick day rules were only known for a minority (1.1%) of drugs for which sick day rules were applicable, whereas for the remaining 98.9% of drugs, patients were unaware that medication adjustment options existed in the event of acute illness.

Comparable to our study, Okuyan and colleagues detected an association between higher age and lower medication knowledge [19]. It might be speculated that patients aged ≥ 80 years in our study generally displayed reduced cognitive functions compared to their younger counterparts, and that this might have been the underlying reason for lower medication knowledge scores. However, even though we did not perform comprehensive neuropsychological testings, cognitive impairment was excluded as part of the clinical evaluation of eligibility. A possible explanation for the lower medication knowledge of patients ≥ 80 years might be delegation of the responsibility for medication preparation and administration to relatives or a nursing service, although this was not investigated in our study. Another explanation might be engagement of patients ≥ 80 years with a healthcare system that has largely been paternalistic and fostered a “doctor knows best” attitude, with fewer opportunities to ask questions and be involved in shared decision making. In contrast to the Okuyan et al. study [19, 20], in which male patients achieved higher medication knowledge scores than female patients, male sex was associated with lower medication knowledge in our investigation.

Although other studies used different methodologies to assess medication knowledge, which substantially limits comparability with our results, it may be assumed that the average medication knowledge in our study was relatively high compared to other investigations (median of 5 for the average medication knowledge score on a scale from 0 to 6). By contrast, in a study by Timmerman et al., which focused on prescriptions of analgesics, around 50% of the study population had no knowledge of at least one aspect (i.e., medication name, dosage, or frequency of application) of their prescription [4]. Similarly, 54.8% of the patients interviewed by Sancar et al. did not know the reason they were taking drugs, and 60.3% did not know when or how to take their medication [21].

In a study by Yasein and co-workers, only 34.8% of the study population showed full concordance between the self-reported number of drugs and the number of drugs in the medical records [22]. The rest of the patients either underestimated (43.4%) or overestimated (21.8%) the number of drugs, without differences between male and female patients [22]. Analogous results were obtained by Zwietering and colleagues, who reported that the number of medications patients stated to use only agreed in approximately 30% to the number of drugs listed in their medication charts [23].

In our study, 95% of patients used their medication charts as support. Freyer et al. found that use of a medication chart is associated with better medication knowledge, and that higher age is associated with less medication knowledge [6], consistent with our results. Freyer and colleagues also noted that medication charts frequently were not up-to-date [6]. Most of the medication charts used by our study participants were professional medication schedules created by patients’ GPs; however, sometimes hospital discharge letters, handwritten tables, (empty) drug packagings with handwritten notes on top, or even smartphone photos were used as “medication charts” by the patients. All of these modalities certainly aided participants in answering our study questionnaire and likely were superior to not using a medication chart at all. Yet, for the purpose of pharmacotherapy safety and a quicker and better overview for attending physicians, it would be desirable—as stipulated in the German eHealth Act (E-Health-Gesetz) from 2015 [24]—to use a nationally standardized (electronic) format for medication charts and to regularly update medication charts.

Despite the overall encouraging results of our study (median of 5 for the average medication knowledge score on a scale from 0 to 6), there is still room for improvement of patients’ medication education, especially among male patients and among patients ≥ 80 years. This is important because the number of German inhabitants aged 85 or older is rapidly growing. Since 1991, their number more than doubled from 1.2 million to 2.6 million by 2021 [25]. This demographic challenge requires enormous efforts by all healthcare workers to safeguard adequate pharmacotherapy and healthcare for older people [22].

In 2008, Modig et al. stated that older people only rarely use the Internet as a source of information about their medication [26], a notion which appears to be corroborated by our study in which only 3% of participants stated that the Internet contributed most to their medication knowledge. Other media such as television and the press were even less represented (0% and 1%, respectively). It is noteworthy that in the media era of the 21^th^ century 69% of participants in our study reported that physicians (GPs but also medical specialists) contributed most to their medication knowledge. Pharmacists, by contrast, apparently did not contribute significantly to patients’ medication knowledge in the present study. It must be noted that we did not specifically explore which source(s) of information participants considered as trustworthy. Older persons’ level of trust in different sources of information should be the subject of future follow-up studies.

The implementation of sick day rules was assessed by NHS Highland for drugs used in the treatment of common diseases of older people (e.g., arterial hypertension, chronic heart failure, diabetes mellitus type 2) [14, 15] but also more specifically for patients with endocrine disorders by Pal and Bhadada [27]. Malik and colleagues issued recommendations for adjusting medication regimens for patients with chronic kidney disease who fast during Ramadan [28]. The cited studies not only emphasized the usefulness of sick day rules, but also made suggestions on how, when, and by whom sick day rules should be communicated to patients [14, 15, 27, 28]. By contrast, only few studies investigated patients’ awareness of sick day rules. Salehmohamed et al. reported that knowledge of sick day rules may differ between patients being treated with the same class of medication (in their study corticosteroids) but for different indications [8]. Our findings reveal major knowledge deficits of older patients regarding options for medication adjustment during episodes of acute illness. To the best of our knowledge, physicians’ awareness of sick day rules has not been investigated to date but should be assessed in future studies as it might significantly influence patients’ awareness of sick day rules.

Older patients are generally more susceptible to adverse effects of drugs due to altered pharmacokinetics and pharmacodynamics [29]. Potentially life-threatening events such as acute kidney injury might be prevented if drugs such as diuretics, angiotensin-converting enzyme inhibitors, angiotensin receptor blockers, or non-steroidal anti-inflammatory drugs are temporarily discontinued during intercurrent illness, but also during radiological or surgical procedures, although the evidence base for this recommendation is limited [11, 30]. Nonetheless, sick-day rules have been widely propagated for use in primary care by NHS Scotland and the Scottish Patient Safety Programme [13–15].

Although Morris et al. reported that patients who were actively engaged in their medication management expressed greater confidence [10], standardized strategies to inform patients about sick day rules do not exist to date. Morris et al. stated that simply handing out a sick day rule plan to patients was insufficient and saw the need for a “wider, collective, [sic] commitment” and for a “clarity in roles and responsibilities” in the future [10]. It is of paramount importance to define who will take over responsibility for this task and at what timepoint in the medication process [10].

Possible ways to make patients aware of the need of medication adjustment are IT system reminders [10], MedicAlert bracelets, or awareness cards, which are already used by some patients treated with corticosteroids in Ireland [8]. In the Morris et al. study, applying highlighted stickers on the back of drug packagings of drugs for which sick day rules are applicable, together with 5-min briefings, was suggested as a possible solution, but also judged as too time-consuming as this would be necessary for every patient taking drugs that can be adjusted during acute illness [10].

Limitations of our study mainly arise from its monocentric setting and the relatively small number of patients enrolled, limiting the generalizability and transferability of our results to other healthcare settings and geographical regions. Although the questionnaire used in our study was not formally validated, a pretest was conducted to ensure the study questionnaire’s applicability under real-world conditions in a hospital setting.

In conclusion, our study showed that medication knowledge of older patients was overall satisfying. Use of a medication chart may have contributed substantially to the sound medication knowledge in our study population. Awareness of sick day rules, however, was poor. Future studies should evaluate the clinical benefits of sick day rules and ways of better communicating sick day rules to patients. In this regard, general practitioners may play a decisive role.

## Supplementary Information

Below is the link to the electronic supplementary material.

Supplementary file1 (DOCX 60 KB)

Supplementary file2 (DOCX 60 KB)

## Data Availability

The data that support the findings of this study are available upon reasonable request from the corresponding author.
