# Supplementary material for: What do older patients know about their medication? A cross-sectional, interview-based pilot study
Source: Eur J Clin Pharmacol. 2023 Aug 10;79(10):1365–74. doi: 10.1007/s00228-023-03548-7 (PMC10501933; doi:10.1007/s00228-023-03548-7)
Supplement: Supplementary file 1 — Supplementary file1 (DOCX 60 KB) [file 228_2023_3548_MOESM1_ESM.docx]

**Questionnaire about medication knowledge of older patients**

**Date: Patient ID: Was a medication plan used during the interview?** □ Yes □ No

| **Drug no.** | What is the name of the drug? | | Is the indication of the drug known? | | | What is the dose of the drug? | | | How frequently is the drug taken? | | Is there anything to observe for this drug in case of acute illness? | | |
| --- | --- | --- | --- | --- | --- | --- | --- | --- | --- | --- | --- | --- | --- |
|  | 0 | 1 | 0 | 1 | 2 | 0 | 1 | 2 | 0 | 1 | 0 | 1 |  |
|  |  | |  | | |  | | |  | |  | | |
|  |  | |  | | |  | | |  | |  | | |
|  |  | |  | | |  | | |  | |  | | |
|  |  | |  | | |  | | |  | |  | | |
|  |  | |  | | |  | | |  | |  | | |
|  |  | |  | | |  | | |  | |  | | |
|  |  | |  | | |  | | |  | |  | | |
|  |  | |  | | |  | | |  | |  | | |
|  |  | |  | | |  | | |  | |  | | |
|  |  | |  | | |  | | |  | |  | | |

How would you evaluate the number of drugs you are taking per day?

| 1 = too few | 2 = rather too few | 3 = adequate number | 4 = rather too many | 5 = too many |
| --- | --- | --- | --- | --- |

Who has contributed the most to your medication knowledge/From whom do you receive the most information about your medication? (single choice)

| O Pharmacy | O Television |
| --- | --- |
| O General practitioner | O The press, magazines |
| O Medical specialist | O Internet |
| O Partner/spouse, relatives, friends | O Other: |

**Notes during the interview**

___________________________________________________________________________________________________________________________________________________________________________________________________________________________________________________________________________________________________________________________________________________________________________________________________________________________________________________________________________________________________________________________________________________________________________________________________________________________________________________________________________________________________________________________________________________________________________________________________________________________________________________________________________________________________________________________________________________________________________________________________________________________________________________________________________________________________________________________________________________________________________
