# Supplementary material for: What do older patients know about their medication? A cross-sectional, interview-based pilot study
Source: Eur J Clin Pharmacol. 2023 Aug 10;79(10):1365–74. doi: 10.1007/s00228-023-03548-7 (PMC10501933; doi:10.1007/s00228-023-03548-7)
Supplement: Supplementary file 2 — Supplementary file2 (DOCX 60 KB) [file 228_2023_3548_MOESM2_ESM.docx]

**Fragebogen zum Medikationswissen älterer Patient*innen**

**Datum: Patient*innen-ID: Wurde ein Medikationsplan zur Beantwortung genutzt?** □ Ja □ Nein

| **Medikament Nr.** | Wie lautet der Name des Medikaments? | | Ist der Verschreibungs­grund bekannt? | | | In welcher Dosis wird das Medikament eingenommen? | | | Wie häufig wird das Medikament eingenommen? | | Gibt es eine Anpassungsmaßnahme im Krankheitsfall? | | |
| --- | --- | --- | --- | --- | --- | --- | --- | --- | --- | --- | --- | --- | --- |
|  | 0 | 1 | 0 | 1 | 2 | 0 | 1 | 2 | 0 | 1 | 0 | 1 |  |
|  |  | |  | | |  | | |  | |  | | |
|  |  | |  | | |  | | |  | |  | | |
|  |  | |  | | |  | | |  | |  | | |
|  |  | |  | | |  | | |  | |  | | |
|  |  | |  | | |  | | |  | |  | | |
|  |  | |  | | |  | | |  | |  | | |
|  |  | |  | | |  | | |  | |  | | |
|  |  | |  | | |  | | |  | |  | | |
|  |  | |  | | |  | | |  | |  | | |
|  |  | |  | | |  | | |  | |  | | |

Wie schätzen Sie die Anzahl der täglich von Ihnen eingenommenen Medikamente ein?

| 1 = zu wenige | 2 = eher zu wenige | 3 = angemessene Anzahl | 4 = eher zu viele | 5 = zu viele |
| --- | --- | --- | --- | --- |

Wer hat am meisten zu Ihrem Wissen über Ihre Medikamente beigetragen/Von wem erhalten Sie die meisten Informationen über Ihre Medikamente? (Einfachauswahl)

| O Apotheke | O Fernsehprogramm |
| --- | --- |
| O Hausarztpraxis | O Presse, Zeitschriften |
| O Facharztpraxis | O Internet |
| O (Ehe-)Partner, Angehörige, Freunde | O Andere: |

**Notizen während des Interviews**

___________________________________________________________________________________________________________________________________________________________________________________________________________________________________________________________________________________________________________________________________________________________________________________________________________________________________________________________________________________________________________________________________________________________________________________________________________________________________________________________________________________________________________________________________________________________________________________________________________________________________________________________________________________________________________________________________________________________________________________________________________________________________________________________________________________________________________________________________________________________________________
